# And It Wasn’t the Stegomyia Fasciata after All

**Published:** 1907-02

**Authors:** 


### AND IT WASN'T THE STEGOMYIA FASCIATA AFTER ALL.

The mosquito we have chased from the sunny shores of South America to the flowering prairies of Illinois—the insect we have abused, maligned, cursed, smoked-out and swatted—the one we have blamed for yellow fever and various other ills—it wasn't the Stegomyia Fasciata at all but another vicious member of the Stegomyia family. So far as we are able to learn from the United States Bureau of Entomology, whose duty it is to pass upon the pedigrees of various insects, nice and unnice, the Stegomyia Fasciata is a perfectly respectable, law-abiding mosquito that has never been guilty of the slightest violations of the rules of propriety, while the nefarious insect that has caused so much damage in the propagation of yellow fever and has brought such disrepute upon his fair name, is, in reality the Stegomyia Calopus. Nothing could afford us greater pleasure than thus relieving the Stegomyia Fasciata of the odium which has unfairly been placed upon him.—*Chicago Clinic and Pure Water Journal*, January, 1907.

